# Heme and blood-feeding parasites: friends or foes?

**DOI:** 10.1186/1756-3305-3-108

**Published:** 2010-11-18

**Authors:** Shu Qin Toh, Amber Glanfield, Geoffrey N Gobert, Malcolm K Jones

**Affiliations:** 1 Queensland Institute of Medical Research, Herston, Queensland, 4006, Australia; 2 School of Chemistry and Molecular Sciences, The University of Queensland, Queensland, 4072, Australia; 3 School of Veterinary Sciences, The University of Queensland, Queensland, 4072, Australia

## Abstract

Hemoparasites, like malaria and schistosomes, are constantly faced with the challenges of storing and detoxifying large quantities of heme, released from their catabolism of host erythrocytes. Heme is an essential prosthetic group that forms the reactive core of numerous hemoproteins with diverse biological functions. However, due to its reactive nature, it is also a potentially toxic molecule. Thus, the acquisition and detoxification of heme is likely to be paramount for the survival and establishment of parasitism. Understanding the underlying mechanism involved in this interaction could possibly provide potential novel targets for drug and vaccine development, and disease treatment. However, there remains a wide gap in our understanding of these mechanisms. This review summarizes the biological importance of heme for hemoparasite, and the adaptations utilized in its sequestration and detoxification.

## Introduction

Blood is a comprehensive nutrient-rich mixture, consistent in composition and continuously renewable for the life of the host [1,2]. Hematophagy is in one respect, an easy "way-out" for parasites in acquiring essential nutrients for their development and reproduction [1]. In addition to the abundant examples of blood dwelling protozoan parasites, hematophagous species are found frequently in many parasitic invertebrates, most notably the polyopistocotylean monogeneans, many digenean flukes, nematodes, hirudinean annelids, insects, acarines and crustaceans. There are even notable examples within vertebrate groups that include birds, mammals, reptiles and fish.

As red blood cells (RBC) constitute the largest cellular component of blood [2], their efficient lysis and catabolism are central requirements for blood-feeding parasites [3]. Each parasite is equipped with a repertoire of complex cascades of hemolytic and proteolytic enzymes [3-7], characterized by general functional redundancies in substrate specificity [3,5,7,8].

Heme, an essential prosthetic group, is liberated from Hb by hemoglobinolysis [9]. Exogenous (dietary) heme has been implicated as a source of metabolic heme and iron (Fe) in some hematophagous parasites [10-12]. However, for many parasites, the pathways associated with heme uptake and liberation of Fe are poorly understood, if indeed, they occur. While an essential nutrient, heme in its free form is also potentially toxic and its rapid detoxification in sanguinivorous parasites is paramount for their survival.

In this review, the biological importance of heme for hemoparasites, and the adaptations utilized in its sequestration and detoxification are presented. Many authors have highlighted the importance of heme-parasite interaction in survival, development and fecundity of these hematophagus parasites [10,13-17]. As such, understanding the underlying mechanisms involved in this interaction could possibly provide novel targets for drug and vaccine development, and disease treatment.

### Heme

Heme consists of an iron (Fe) atom bound to four nitrogen atoms of the pyrrole ring of protoporphyrin IX. Fe is a transitional metal, existing in either a ferrous (Fe^2+^) or a ferric (Fe^3+^) forms. This transitional property imparts the catalytic nature to many compounds that contain Fe [18]. Porphyrin is an organic compound capable of producing singlet oxygen (^1^O_2_) in its excited state [19]. Due to its hydrophobic nature, porphyrin can interact with lipophilic molecules, including proteins and lipids [19]. The reactive nature of both Fe and porphyrin make heme an essential, yet potentially toxic, molecule [18,19].

Heme forms the reactive core of numerous hemoproteins with diverse biological functions. Heme also interacts with biologically important molecules, either by direct binding, or by transient interaction which lead to free radical generation [19,20]. In binding macromolecules, heme can destabilize cell membranes, resulting in cell lysis [20-22], or it can bind various transcriptional factors and enzymes, thereby modulating protein synthesis transcriptionally and translationally, leading to the regulation of cell development, differentiation, signal transduction and apoptosis [23-25].

Heme can generate both hydroxyl radicals and reactive oxygen species (ROS) and can induce lipid peroxidation [26-28]. The contained Fe is postulated to catalyze the formation of ROS through the Fenton reaction [19,22], although the specific heme-H_2_O_2 _reaction has also been shown to generate free radicals [28]. Heme-induced free radicals are potentially toxic, capable of damaging and degrading proteins, lipids and DNA molecules [29,30]. However, low levels of ROS are essential for many biological functions, including the up-regulation of heme biosynthesis, activation of enzymes (e.g catalase) and signal transduction in cells [24,25].

### Heme Biosynthesis

**Figure 1:**
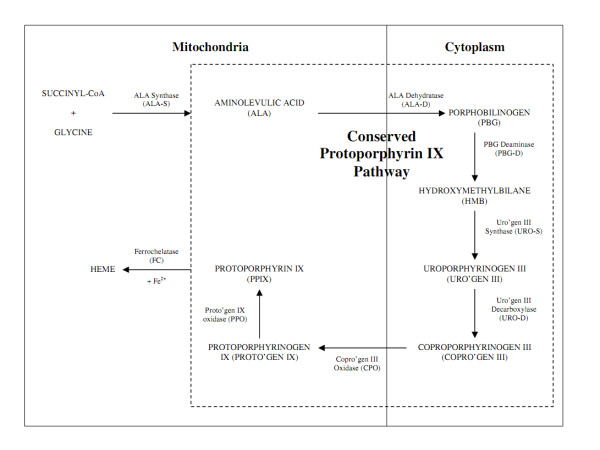
**Heme Biosynthetic Pathway**. Initiation of heme biosynthesis begins in the mitochondria with the condensation of succinyl coenzyme A (CoA) and glycine, catalyzed by ALA synthetase (ALA-S), form δ-aminolevulinic acid (ALA). Conserved biosynthetic protoporphyrin IX (PPIX) pathway: ALA is catalyzed by ALA dehydratase (ALA-D) in the cytoplasm to form porphobilinogen (PBG). Four molecules of PBG are combined by PBG deaminase (PBG-D) to form into the cyclic tetrapyrrole hydroxymethylbilane (HMB) and converted to Uroporphyrinogen III (Uro'gen III) by Uroporphyrinogen III synthase (URO-S). Uro'gen III is converted by Uro'gen III decarboxylase (URO-D) to coproorphyrinogen III (Copro'gen III), with the removal of CO_2_. Subsequent conversion of Copro'gen III to Protoporphyrinogen IX (Proto'gen IX) and finally protoporphyrin IX (PPIX) occurs in the mitochondria. This is catalyzed by the actions of Coproporphyrinogen III Oxidase (CPO) and Protophyrinogen IX Oxidase (PPO) respectively. Finally, ferrous iron (Fe2+) is inserted into the cyclic macrocycle through the action of ferrochelatase (FC) in the mitochondria.

In non-photosynthetic eukaryotes, heme biosynthesis begins with the condensation of succinyl-CoA and glycine, forming δ-aminolevulinic acid (ALA) and subsequent heme biosynthetic pathway involves seven enzymes (Figure 1) [9,31].

### Sources of Parasite Heme

#### Heme Biosynthesis

The evolutionary loss of an endogenous heme biosynthetic pathway is thought to be the concomitant outcome of the adoption of hematophagy in these hemoparasites [32-34]. Complete loss of *de novo *heme biosynthesis has been postulated for the nematodes *Ancylostoma caninum*, *Haemonchus contortus*, *Ascaris suum*, the nematomorph *Paragordius varius *(a parasite in the hemocoele of insects), the digeneans *Schistosoma mansoni *and *Philophthalmus megalurus*, the tick *Rhipicephalus microplus *and the hemoflagellate *Leishmania tarentolae*, losses determined on the basis of the absence of enzymatic activities for ALA-D, PBG-D and FC (See Figure 1) [33,35,36]. Partial loss of the heme biosynthetic ability is postulated for the trypanosomatids *L. amazonenesis*, *L. infantum*, and *Trypanosoma cruzi *and the filariid nematode *Brugia malayi*, in which the first five enzymes of the pathway are thought to be absent, while the latter three are functional [37,38].

#### Scavenging of Host Heme

Although some studies suggest that malaria, the filariid *Setaria digitata *and the triatomid hemipteran, *Rhodnius prolixus*, synthesize heme *de novo *[35,39,40], other studies of malaria and *R. prolixus *indicate that these parasites rely, to a certain extent, on host heme [11,40,41]. For *Plasmodium falciparum *and *P. berghei*, it has been demonstrated that host biosynthetic enzymes present in the RBCs are trafficked into the parasite and account for about 80% of the enzymatic activities of intra-parasite heme biosynthesis [42]. Inhibiting the trafficking of host ALAD leads to death of intrerythrocytic stages of malaria [43].

Evidence for heme uptake pathways has also been found for *Trypanosoma cruzi*, *Leishmania spp *and even for non-hematophagous parasites, like the luminal parasitic cestode *Hymenolepis microstoma *[13,15,44,45]. For these parasites, heme is an absolute nutritional requirement for growth and development, with strong positive correlations between heme availability and parasite growth, survival and reproduction and parasite death in the absence of heme and presence of iron. Uptake of the heme moiety of Hb or heme analogues has also been demonstrated *in vivo *in *Schistosoma mansoni *schistosomula, malaria, the tick *Rhipicephalus microplus *and the triatomine bug *Rhodnius prolixus *[35,40,46,47].

Loss of an endogenous heme biosynthetic pathway in parasites is theorized to have arisen from two contributing factors. Firstly, hematophagy provides an abundance of readily available heme. Secondly, as stated, *de novo *heme biosynthesis is an oxygen-dependent pathway, involving eight enzymes (Figure 1). Many endoparasites are facultative anaerobes and anaerobic metabolism is thought to occur as a counter-measure against oxidative stress from Hb catabolism [48]. Therefore, *de novo *heme biosynthesis might conceivably place undesirable stress on these parasites. Furthermore, loss of an endogenous heme biosynthetic pathway is likely to involve a complex interplay of many factors, including adoption of heteroxenous life cycles, the presence of multiple stages in the life-cycle and the involvement of symbiotic organisms, such as the obligatory endocommensal microbe *Wolbachia*, which synthesises heme utilized by its hosts [33,38,49].

### Heme Uptake and Transport

In all multicellular organisms, an efficient set of pathways to circulate heme is essential to ensure that the 'heme pool' is maintained at physiological level [9]. In these organisms, heme is transported from the extracellular-to-intracellular environment and from cytoplasm to mitochondria, and is "scavenging" by molecules when found in its free state. Efficient transmembrane transport of heme is crucial in supporting biological reactions and hemoprotein synthesis [50,51].

#### Heme Transporter Proteins (HTPs)

Although Light and Olson (1990) demonstrated that free heme can diffuse across membranes, they showed that passive diffusion is too slow to efficiently support essential biological reactions as well as hemoprotein synthesis. Furthermore, heme binding by cells is a saturable, temperature-dependent, tissue-specific and reversible process [52]. Heme uptake is regulated by factors like hypoxia [53]. Given the potential toxicity of heme, transmembrane movement of heme is likely to be an energy-dependent activity mediated by HTPs. Four HTPs have been identified for mammals, namely, the heme carrier protein (HCP1) [53], ATP-binding cassette subgroup B member 6 [54], feline carrier leukemia receptor C and the ATP-binding cassette subgroup G member 2 [55]. Information on the exact mechanisms involved in the transmembrane movement of heme is limited, although it has been suggested to involve specific binding of heme or heme-bound complexes to the membrane surface, followed by active transmembrane transportation possibly through a proton pump [53].

In hematophagus parasites, information on these mechanisms is even scarcer, despite numerous studies showing the utilization and recycling of exogenous heme source (e.g. catabolism of host RBC) as essential for growth and development as well as more specifically embryogenesis. In *Leishmania. m. amazonenesis *[56] and *T. cruzi *[57], heme uptake is saturable and specific. For *T. cruzi*, heme uptake may involve the presence of an ATP-binding cassette (ABC) transporter [57], a postulate supported by an inhibition assay of heme uptake, in which accumulation of the fluorescent heme analogue palladium mesoporphyrin was detected at the surface of cells after pre-incubation of parasites with cyclosporin A, an inhibitor of ABC transporters including P-glycoproteins (PgP) [57]. Since studies of *Leishmania *have demonstrated the lack of a functional heme biosynthetic enzymatic pathway [13,15,44,45], exogenous heme transported through HTPs could also be a source of heme for these parasites.

#### Heme Binding Proteins (HBPs)

Due to its reactive nature, heme is usually bound to protein in circulation, and is rarely found as a free molecule. HBPs are important components for heme detoxification and for recycling of heme and Fe, since they allow the targeting of heme to specific tissues such as the liver [58,59], while protecting other tissues against heme-mediated damage [58,60]. In humans, this sequestration and recycling of circulating heme and Hb during intravascular hemolysis and tissue damage, has been attributed to three main heme binding proteins: haptoglobin [59], hemopexin and albumin [58].

For blood-feeding parasites, catabolism of host RBC results in the release of large quantities of heme. This concentration of dietary heme represents a condition unlike that observed for mammals, in which heme abundance is tightly regulated. Parasitic arthropods, including the ticks, mosquitoes and hemipterans [61-63] express HBPs.

Two HBPs of ixodid and argasid ticks are hemelipoglyco-carrier protein (CP) and vitellogenin (Vg) [61,64]. CP and VG are similar in structure, binding affinity to heme, lipid and carbohydrates, and in signaling cascades leading to their expression during blood-feeding, but have distinct functions, tissue distribution, and regulatory controls [64]. CP (also known as HeLp in *Rhipicephalus microplus*) [65] is a major hemolymph protein composed of two subunits, with molecular mass ranging from 200 to 500kDa [64]. CP is detected in both male and female parasites, is highly expressed in the fat bodies and salivary glands and is up-regulated in response to blood-feeding [66]. CP is thought to sequester heme and subsequently transfer it to the hemocoel [61,66], for subsequent transfer to other cytoplasmic HBPs to meet the heme needs of these parasites [67]. Vg, a precursor of the large multimeric yolk protein, is found primarily in the female tick and egg, although it is synthesized by fat bodies [4,64,66]. Vg is induced by mating and increased levels of ecdysteroid, is secreted into the hemolymph and then transported and incorporated into developing oocytes by receptor mediated endocytosis [61,66].

A 15kDa heme-binding protein (RHBP), which shares similar functions with Vg of ticks, has been isolated from both the hemolymph and oocytes of *Rhodnius prolixus *[68]. As with Vg, RHBP is postulated to deliver heme to developing oocytes for vitellogenesis and embryogenesis [61,68].

While knowledge of HBPs in arthropods is accumulating, the data on HBPs of helminth parasites is scant. Annotated screening of genomic datasets of *S. mansoni *[69] reveal a putative heme-binding protein, which shares a conserved tertiary fold structure with members of the SOUL/HBP superfamily [70]. Transcription of this schistosome molecule is increased 3-4-fold in schistosomules cultured in the presence of RBC compared with those cultured in media alone [71]. The elevated transcription of a molecule bearing structural similarity with known heme transporters in the presence of Hb suggests that this helminth also expresses functional HBPs.

### Heme Detoxification

**Figure 2:**
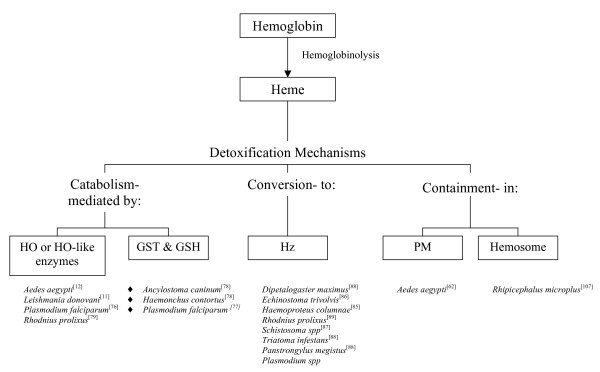
**Summary of the known and possible heme detoxification mechanisms in hematophagous parasites, based on data published in references **[11,12,62,76-79,86-89,107]. Abbreviations: HO, heme oxygenase; GST, glutathione-S-transferase; GSH, reduced glutathione; Hz, hemozoin; PM, peritrophic matrix.

The requirement to excrete and detoxify the bulk of ingested heme is essential for hematophagous organisms. This can be demonstrated in the *in vitro *culture of *L. donovani*, in which elevated heme concentrations results in parasite death [45]. Moreover, the success of anti-parasitic compounds, such as the quinolines and artemether, both of which are postulated to inhibit heme detoxification pathways in malaria and schistosomes [72-74], is strong evidence for the importance of this pathway. Numerous detoxification mechanisms have been identified in parasitic organisms (Figure 2), including the breaking down of heme into Fe and less reactive intermediates, the containment of heme by a physical barrier, and converting heme into an inert crystalline structure.

#### Heme Oxygenase

In the presence of oxygen and NADPH, human heme oxygenase (HO) catalyzes the oxidation of heme to α-meso-hydroxyheme, verdoheme and finally, biliverdin. Biliverdin is further metabolized to bilirubin and excreted in the bile, conjugated with glucuronic acid [75]. Similar catalytic mechanisms by HO-like proteins are postulated to also occur in bacteria and plants [76]. Conclusive evidence for HO-like activity in haematophagous parasites is lacking, although there has been recent reports indicating that HO-like activity does occur [11,77]. The absence of HO homologues in parasitic organisms have led some researchers to postulate alternative pathways for heme catabolism and Fe rescue [78,79].

Injection of excess heme into the hemocoel of *R. prolixus *results in the production of a biliverdin-like compound (RpBV), which differs from biliverdin in possessing two additional cysteine residues [80], an observation that strongly suggests HO-like activity in this hemipteran [80]. Similarly, HO-like catabolic system is thought to exist in *Aedes aeypti*, as it has been shown that most Fe in adult mosquitoes are derived from host heme [12].

Recently, a *P. falciparum *HO (PfHO), which shares similar heme catabolic activity with mammalian HO despite limited amino acid sequence identity, has been identified [77]. PfHO is similar to plastidial HO of cyanobacteria, algae and other higher plants, all of which use reduced ferredoxin as its electron source, instead of NADPH cytochrome P450 reductase as the mammalian HO does [77]. HO-like activity has also been demonstrated for *P. berghei *and it has been noted that significantly higher HO activities were detected in chloroquine-resistant strains [81].

#### Glutathione-S-Transferase (GST) & Reduced Glutathione (GSH)

Members of the glutathione-S-transferase (GST) family characteristically catalyze the breakdown of endogenous and exogenous toxins with a demonstrated heme catabolic activity found in reduced glutathione (GSH) [82]. Multiple heme-responsive GSTs with high affinity binding to heme have been identified in *H. contortus *and *A caninum *using sub-proteomic approaches and it has been suggested that GST is important for transport and detoxification of heme [78,79].

Fueled by reports indicating a correlation between chloroquine (CLQ) resistance in malaria with a loss of hemozoin formation [83], it has been proposed that other heme detoxification mechanisms are present in resistant parasites. GST and GSH are thought to be important components in the alternative detoxification of heme in malaria [78,79,83], since increased GST and GSH activities [81,83,84] occurs in CLQ-resistant strains. It is possible that malaria GST acts by binding heme and conjugating it to GSH [78]. GSH, through a series of reactions in the presence of oxygen, produces free radicals that cleave heme at the porphyrin ring, releasing Fe [82]. There remain questions as to how this mechanism supports heme detoxification in these parasites, including how they deal with the free radicals and how Fe is generated. Furthermore, human GST (hGST) is only active at pH > 6.5 [82] whereas the optimum pH of *P. falciparum *GST (PfGST) is 8.8 [85]. However, the digestive vacuoles of the malaria parasite are acidic [14], indicating GST and GSH activity may not occur in that compartment, which is the site of heme release.

#### Hemozoin (Hz)

**Figure 3:**
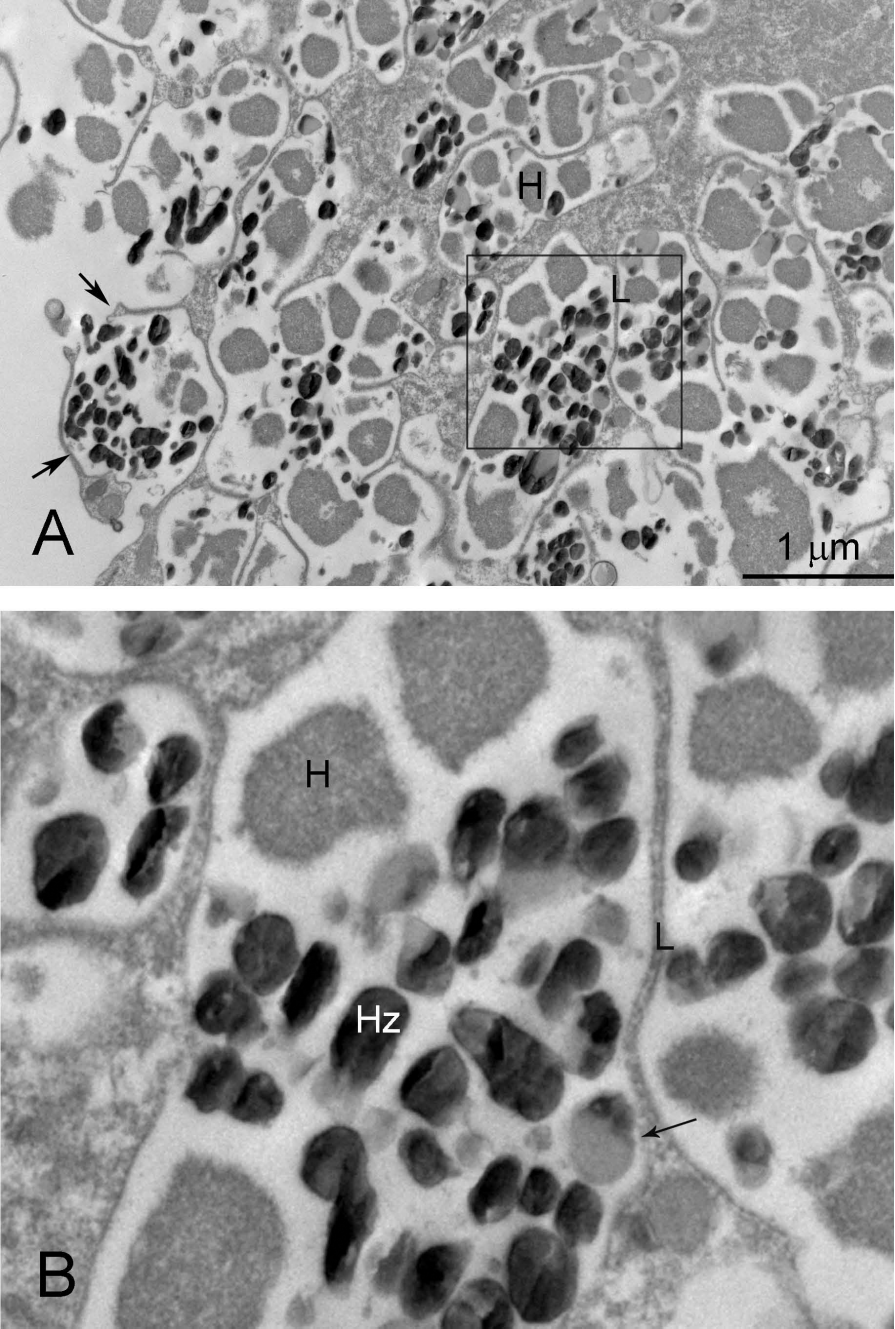
**Transmission Electron Microscope image of the gastrodermis (syncytial absorptive gut lining) of an adult female *Schistosoma japonicum***. The parasite was fixed in 3% glutaraldehyde in cacodylate buffer supplemented with 5% tannic acid, followed by fixation in osmium tetroxide and embedded in Spurr's resin. Hemozoin formation in the schistosome gut occurs in structure believed to be epicellular vacuolar compartments (or luminal pockets of the gastrodermis-see Delcroix *et al*. [8]) formed by extensive growth of surface lamellae of the syncytial lining. A. Micrograph of apical region of the gastrodermal syncytium showing abundant epicellular vacuolar compartments (arrows). The pale bodies (H) in the lumens of the vacuoles are regions of hemoglobin. The dark bodies (Hz) are hemozoin crystals. B. Enlargement of boxed region of A. Hemozoin forms at the surface of lipid particles. Abbreviations: H-fragment of erythrocyte; Hz-hemozoin; L-lamella of gastrodermis.

Hz formation is a heme detoxification mechanism observed only in hematophagus parasites. It is interesting that Hz formation is found in divergent groups of parasites including malaria, schistosomes (Figure 3) and some insects.

Subsequent to the initial association of the pigment with malaria, this detoxification mechanism has also been described for the protistan *Haemoproteus columnae *[86], the digeneans *Echinostoma trivolvis *and *Schistosoma *[87,88] and triatomine bugs [89,90]. The Hz polymerization pathway is proposed to be an excellent therapeutic target for malaria and other parasites [72-74]. The antimalarial quinoline, for example, limits intra-erythrocytic parasite growth by inhibiting Hz formation [72]. Similarly, quinoline inhibits Hz formation in *S. mansoni in vivo *and *in vitro *[73,91].

Hz is single unit polymer of heme [92,93]. Based on its physical and molecular properties, Hz differs from heme aggregates, but is similar to the synthetic β-hematin [94]. Unlike heme aggregates, Hz is insoluble in bicarbonate buffer and SDS [95] and shows the two characteristic peaks at 1,209 and 1,662 cm^-1 ^with absence of the characteristic heme peak at 1,707 cm^-1 ^by FTIR spectra [96]. The structure of Hz has been confirmed by X-ray diffraction [97], infrared spectroscopy and extended absorption fine structure (EXAFS) [94,98] as a lattice of hydrogen-bonded heme dimers, linked in a head-to-tail manner via iron-carboxylate bond with the ferric iron of one heme unit bound to the propionate side chain of another heme unit.

The mechanisms of Hz formation among different parasites remain unresolved. Proteins, lipids, alcohol, acids and even an autocatalytic mechanism have been proposed to bring about the efficient conversion of heme to Hz. Most recently, neutral lipids have been implicated as the primary catalysts [99] causing heme to be converted to β-hematin at the lipid surface by interaction with lipid polar heads. Growth of the β-hematin crystal appears limited by the curvature of the lipid particle [99].

Protein was once thought to facilitate the polymerization of Hz [100], although this hypothesis has more recently been discounted [101]. It is now thought that proteins function as initiation sites for the primary nucleation step or as chaperones for delivery of heme to the lipid nanosphere through high affinity binding to heme [102]. Two proteins, a histidine-rich protein (HRP) and heme detoxification protein (HDP) have been identified in *Plasmodium *spp as potential initiation sites for Hz formation. These histidine-rich proteins bind with high affinity to heme, indicating possible bis-histidyl heme iron coordination [103,4] . However, the involvement of HRP as an essential catalytic component of Hz formation has been questioned, because Hz conversion has been noted in some malaria species in the absence of a functional HRP [105]. On the other hand, malaria HDP is able to mediate high levels of heme conversion, is highly conserved across the *Plasmodium *genus, and is internalized into food vacuoles of the parasite, suggesting a role in Hz formation [104].

### Heme Containment

#### Peritrophic Matrix (PM)

An efficient means used by some blood-feeding parasites to separate heme is by the use of a physical barrier, as seen ion PMs. These structures line the mid-gut of invertebrates and are synthesized by one or more stages of the majority of insects examined [105]. PMs are semi-permeable structures that encase an ingested meal, partitioning it from the gut epithelial cell lining while retaining selective permeability to derived compounds and nutrients [106]. PMs are complex matrices, composed of proteins, proteoglycans and chitins [105], and are thought to protect the intestinal epithelium against damage caused by the abrasion of food particles [106]. In hematophagous arthropods, PMs may also protect against damage from toxic metabolites [106], as well as against invasion by viruses and bacteria [105].

Heme binds specifically to the PM of *Ae. aegypti *[62], suggesting a possible role for the matrix in heme containment. Such binding is thought to be augmented by *Ae. aegypti *intestinal mucin (AeIMUCI), which has been isolated from both the larvae and adults [107]. Expression of AeIMUCI is induced by metal feeding and the protein can bind to chitin as well as large amounts of heme *in vitro *[107]. Taken together, these data suggest that the role of the PM in heme detoxification may be more than just that of a passive physical barrier.

#### Hemosomes

An intriguing heme detoxification pathway has been identified for the tick *Rhipicephalus microplus*, in which heme is accumulated as a non-crystalline aggregate in a specialized organelle of midgut epithelium, the hemosome [108]. FTIR spectroscopy of hemosomes revealed that the heme aggregate is distinct from hemozoin and is structurally bonded by a novel heme-based interaction with non-heme components in the hemosome [108]. A mature hemosome consists of a compact centre, composed of approximately 90% heme aggregates, surrounded by a multi-layered cortex, surrounded in turn by a lipid bilayer [108]. Heme is transported from the digestive vacuoles of mid-gut epithelial cells into hemosomes [108]. The exact mechanism facilitating this is unknown, but may involve the use of heme transporters or binding proteins [10]. At completion of digestion of the blood meal and maturation of the hemosomes, the digestive cells detach from the gut wall and are subsequently excreted from the tick with feces [108].

## Conclusions

The reactive nature of heme renders it both a biologically important molecule as well as a toxic compound. In hematophagy, the acquisition and detoxification of heme is paramount for the survival and establishment of parasitism. This is reflected in the fact that parasites are constantly challenged with the need to process huge quantities of heme, released from their catabolism of host erythrocytes. However, there remain many questions surrounding heme metabolism in these organisms:

1. What is the source of heme for many of these parasites? Is there an alternative heme biosynthetic pathway present in these parasites?

2. What are the underlying mechanisms involved in the targeting and uptake of heme in these parasites?

3. What is the importance of heme as an iron source and what enzymatic reactions are involved in catalyzing the release of iron? Are strategies for heme acquisition and distribution completely distinct, or are there overlapping activities [67].

4. What other novel heme detoxification methods are utilized by these parasites?

5. Do helminths express novel HBPs for cytoplasmic storage and intercellular transport of heme?

Despite agreement that pathways of heme biosynthesis, uptake, detoxification and even breakdown might provide potential important targets for drugs and vaccine development, there remain wide gaps in our understanding of the molecular regulation of these events. In helminths the available information regarding parasite-heme interactions remains limited. This is perhaps due to the absence of easily amenable life cycles and inherent difficulties in culturing the different life cycle stages in these parasites. There is thus great need for further work on adaptations of helminths for heme and iron metabolism [109].

## Abbreviations

AeIMUCI: *Ae. aegypti *intestinal mucin 1; ALA: δ-aminolevulinic acid; BV- biliverdin; CLQ: chloroquine; CP: hemelipoglyco-carrier protein; Fe- iron; FTIR: Fourier transform infra-red; GSH: Reduced Glutathione; GST: Glutathione-S-transferase; H_2_O_2 _-hydrogen peroxide; Hb- hemoglobin; HBP: Heme Binding Proteins; HO: heme oxygenase; HTP: Heme Transporter Proteins; SDS: sodium dodeca-sulphate; RBC: red blood cells; ROS: reactive oxygen species; Vg: vitellogenin.

## Competing interests

The authors declare that they have no competing interests.

## Authors' contributions

SQT: Wrote drafts, contributed to design of review AG, GNG, MKJ: Critical review of manuscript; contribution to conception and design. All authors approve of this version of the manuscript and agree to its publication.

## References

[B1] Dalton JP Skelly P Halton DW Role of the tegument and gut in nutrient uptake by parasitic platyhelminths Can J Zool 2004 82 211 232 10.1139/z03-213

[B2] Whitfield PJ The biology of parasitism: an introduction to the study of associating organisms 1979 London: Edward Arnold

[B3] Brindley PJ Kalinna BH Dalton JP Day SR Wong JYM Smythe ML McManus DP Proteolytic degradation of host hemoglobin by schistosomes Mol Biochem Parasitol 1997 89 1 9 10.1016/S0166-6851(97)00098-4 9297696

[B4] Delcroix M Sajid M Caffrey CR Lim KC Dvorak J Hsieh I Bahgat M Dissous C McKerrow JH A multienzyme network functions in intestinal protein digestion by a platyhelminth parasite J Biol Chem 2006 281 39316 39329 10.1074/jbc.M607128200 17028179

[B5] Goldberg DE Plasmodial hemoglobin degradation-an ordered pathway in a specialized organelle Infect Agents Dis 1992 1 207 211 1365547

[B6] Horn M Nussbaumerova M Sanda M Kovarova Z Srba J Franta Z Sojka D Bogyo M Caffrey CR Kopacek P Mares M Hemoglobin digestion in blood-feeding ticks: mapping a multipeptidase pathway by functional proteomics Chem Biol 2009 16 1053 1063 10.1016/j.chembiol.2009.09.009 19875079 PMC2801564

[B7] Rosenthal PJ Meshnick SR Hemoglobin catabolism and iron utilization by malaria parasites Mol Biochem Parasitol 1996 83 131 139 10.1016/S0166-6851(96)02763-6 9027746

[B8] Delcroix M Medzihradsky K Caffrey CR Fetter RD McKerrow JH Proteomic analysis of adult *S. mansoni *gut contents Mol Biochem Parasitol 2007 154 95 97 10.1016/j.molbiopara.2007.03.008 17451823 PMC2732360

[B9] Ponka P Cell biology of heme Am J Med Sci 1999 318 241 256 10.1097/00000441-199910000-00004 10522552

[B10] Lara FA Lins U Bechara GH Oliveira PL Tracing heme in a living cell: hemoglobin degradation and heme traffic in digest cells of the cattle tick *Boophilus microplus* J Exp Biol 2005 208 3093 3101 10.1242/jeb.01749 16081607

[B11] Taylor MC Kelly JM Iron metabolism in trypanosomatids, and its crucial role in infection Parasitology 2010 137 899 917 10.1017/S0031182009991880 20152063

[B12] Zhou GL Kohlhepp P Geiser D Frasquillo MDC Vazquez-Moreno L Winzerling JJ Fate of blood meal iron in mosquitoes J Insect Physiol 2007 53 1169 1178 10.1016/j.jinsphys.2007.06.009 17689557 PMC2329577

[B13] Ciccarelli A Araujo L Batlle A Lombardo E Effect of haemin on growth, protein content and the antioxidant defence system in *Trypanosoma cruzi* Parasitology 2007 134 959 965 10.1017/S0031182007002399 17316475

[B14] Egan TJ Haemozoin formation Mol Biochem Parasitol 2008 157 127 136 10.1016/j.molbiopara.2007.11.005 18083247

[B15] Gaughan PLZ Krassner SM Hemin deprivation in culture stages of hemoflagellate, *Leishmania tarentolae* Compe Biochem Physiol 1971 39 5 10.1016/0305-0491(71)90247-1

[B16] Graca-Souza AV Maya-Monteiro C Paiva-Silva GO Braz GRC Paes MC Sorgine MHF Oliveira MF Oliveira PL Adaptations against heme toxicity in blood-feeding arthropods Insect Biochem Mol Biol 2006 36 322 335 10.1016/j.ibmb.2006.01.009 16551546

[B17] Zhan B Liu S Perally S Xue J Fujiwara R Brophy P Xiao S Liu Y Feng J Williamson A Wang Y Bueno LL Mendez S Goud G Bethony JM Hawdon JM Loukas A Jones K Hotez PJ Biochemical characterization and vaccine potential of a heme-binding glutathione transferase from the adult hookworm *Ancylostoma caninum* Infect Immun 2005 73 6903 6911 10.1128/IAI.73.10.6903-6911.2005 16177370 PMC1230892

[B18] Kehrer JP The Haber-Weiss reaction and mechanisms of toxicity Toxicology 2000 149 43 50 10.1016/S0300-483X(00)00231-6 10963860

[B19] Vincent SH Oxidative effects of heme and porphyrins on proteins and lipids Semin Hematol 1989 26 105 113 2658086

[B20] Chou AC Fitch CD Mechanism of hemolysis induced by ferriprotoporphyrin IX J Clin Invest 1981 68 672 677 10.1172/JCI110302 7276166 PMC370848

[B21] Kirschnerzilber I Rabizadeh E Shaklai N The interaction of hemin and bilirubin with the human red-cell membrane Biochim Biophys Acta 1982 690 20 30 10.1016/0005-2736(82)90234-6 7126567

[B22] Shinar E Rachmilewitz EA Oxidative denaturation of red-blood-cells in thalassemia Semin Hematol 1990 27 70 82 2405497

[B23] Hou S Reynolds MF Horrigan FT Heinemann SH Hoshi T Reversible binding of heme to proteins in cellular signal transduction Acc Chem Res 2006 39 918 924 10.1021/ar040020w 17176030

[B24] Tsiftsoglou AS Tsamadou AI Papadopoulou LC Heme as key regulator of major mammalian cellular functions: Molecular, cellular, and pharmacological aspects Pharmaco Ther 2006 111 327 345 10.1016/j.pharmthera.2005.10.017 16513178

[B25] Ye W Zhang L Heme controls the expression of cell cycle regulators and cell growth in HeLa cells Biochem Biophys Res Commun 2004 315 546 554 10.1016/j.bbrc.2004.01.092 14975735

[B26] Klouche K Morena M Canaud B Descomps B Beraud JJ Cristol JP Mechanism of in vitro heme-induced LDL oxidation: effects of antioxidants Eur J Clin Invest 2004 34 619 625 10.1111/j.1365-2362.2004.01395.x 15379761

[B27] Sadrzadeh SMH Anderson DK Panter SS Hallaway PE Eaton JW Hemoglobin potentiates central nervous system damage J Clin Invest 1987 79 662 664 10.1172/JCI112865 3027133 PMC424162

[B28] Tappel AL Unsaturated lipide oxidation catalyzed by hematin compounds J Biol Chem 1955 217 721 733 13271434

[B29] Aft RL Mueller GC Hemin-mediated DNA strand scission J Biol Chem 1983 258 2069 2072 6619154

[B30] Aft RL Mueller GC Hemin-mediated oxidative-degradation of proteins J Biol Chem 1984 259 301 305 6323403

[B31] Heinemann IU Jahn M Jahn D The biochemistry of heme biosynthesis Arch Biochem Biophys 2008 474 238 251 10.1016/j.abb.2008.02.015 18314007

[B32] Cain GD The source of hemoglobin in *Philophthalmus megalurus *and *Fasciolopsis buski *(*Trematoda *- digenea) J Parasitol 1969 55 307 10 10.2307/3277395 5778807

[B33] Chang KP Chang CS Sassa S Heme biosynthesis in bacterium-protozoon symbioses-enzymic defects in host hemoflagellates and complemental role of their intracellular symbiotes Proc Natl Acad Sci USA 1975 72 2979 2983 10.1073/pnas.72.8.2979 810795 PMC432902

[B34] Ohagan JE *Boophilus microplus *- digestion of hemoglobins by engorged female tick Exp Parasitol 1974 35 110 118 10.1016/0014-4894(74)90013-7 4815010

[B35] Braz GRC Coelho HSL Masuda H Oliveira PL A missing metabolic pathway in the cattle tick *Boophilus microplus* Curr Biol 1999 9 703 706 10.1016/S0960-9822(99)80312-1 10395540

[B36] Rao A Yeleswarapu J Srinivasan R Bulusu G Localization of heme biosynthesis pathway enzymes in *Plasmodium falciparum* Indian J Biochem Biophys 2008 45 365 373 19239121

[B37] Dutta S Furuyama K Sassa S Chang KP *Leishmania spp*.: delta-aminolevulinate-inducible neogenesis of porphyria by genetic complementation of incomplete heme biosynthesis pathway Exp Parasitol 2008 118 629 636 10.1016/j.exppara.2007.11.013 18164705 PMC2423932

[B38] Wu B Novelli J Foster J Vaisvila R Conway L Ingram J Ganatra M Rao AU Hamza I Slatko B The Heme Biosynthetic Pathway of the Obligate *Wolbachia *Endosymbiont of *Brugia malayi *as a Potential Anti-filarial Drug Target PLoS Negl Trop Dis 2009 3 7 e475 10.1371/journal.pntd.0000475 19597542 PMC2703803

[B39] Kumar RA Raj RK Presence and formation of heme and occurrence of certain heme proteins in the filarial parasite *Setaria digitata* Biochem Biophys Res Commun 1998 253 49 52 10.1006/bbrc.1998.9655 9875218

[B40] Surolia N Padmanaban G *De novo *biosynthesis of heme offers a new chemotherapeutic target in the human malarial parasite Biochem Biophys Res Commun 1992 187 744 750 10.1016/0006-291X(92)91258-R 1356337

[B41] Dansapetretski M Ribeiro JMC Atella GC Masuda H Oliveira PL Antioxidant role of *Rhodnius prolixus *heme-binding protein-protection against heme-induced lipid peroxidation J Biol Chem 1995 270 10893 10896 10.1074/jbc.270.18.10893 7738029

[B42] Bonday ZQ Taketani S Gupta PD Padmanaban G Heme biosynthesis by the malarial parasite. Import of delta-aminolevulinate dehydrase from the host red cell J Biol Chem 1997 272 21839 21846 10.1074/jbc.272.35.21839 9268315

[B43] Padmanaban G Nagaraj VA Rangarajan PN An alternative model for heme biosynthesis in the malarial parasite Trends Biochem Sci 2007 32 443 449 10.1016/j.tibs.2007.09.005 17928230

[B44] Chang CS Chang KP Heme requirement and acquisition by extracellular and intracellular stages of *Leishmania mexicana amazonensis* Mol Biochem Parasitol 1985 16 267 276 10.1016/0166-6851(85)90069-6 4058483

[B45] Pal JK Joshi-Purandare M Dose-dependent differential effect of hemin on protein synthesis and cell proliferation in *Leishmania donovani *promastigotes cultured in vitro J Biosci 2001 26 225 231 10.1007/BF02703646 11426058

[B46] Braz GRC Abreu L Masuda H Oliveira PL Heme biosynthesis and oogenesis in the blood-sucking bug, *Rhodnius prolixus* Insect Biochem Mol Biol 2001 31 359 364 10.1016/S0965-1748(00)00129-6 11222945

[B47] Foster LA Bogitsh BJ Utilization of the heme moiety of hemoglobin by *Schistosoma mansoni *schistosomules *in vitro* J Parasitol 1986 72 669 676 10.2307/3281454 3806318

[B48] Oliveira PL Oliveira MF Vampires, Pasteur and reactive oxygen species-Is the switch from aerobic to anaerobic metabolism a preventive antioxidant defence in blood-feeding parasites? FEBS Lett 2002 525 3 6 10.1016/S0014-5793(02)03026-0 12163151

[B49] Koreny L Lukes J Obornik M Evolution of the haem synthetic pathway in kinetoplastid flagellates: An essential pathway that is not essential after all? Int J Parasitol 2009 40 149 156 10.1016/j.ijpara.2009.11.007 19968994

[B50] Galbraith RA Sassa S Kappas A Heme binding to murine erythroleukemia-cells-evidence for a heme receptor J Biol Chem 1985 260 2198 2202 2995365

[B51] Light WR Olson JS Transmembrane movement of heme J Biol Chem 1990 265 15623 15631 2394740

[B52] Worthington MT Cohn SM Miller SK Luo RQ Berg CL Characterization of a human plasma membrane heme transporter in intestinal and hepatocyte cell lines Am J Physiol Gastrointest Liver Physiol 2001 280 G1172 G1177 11352810 10.1152/ajpgi.2001.280.6.G1172

[B53] Shayeghi M Latunde-Dada GO Oakhill JS Laftah AH Takeuchi K Halliday N Khan Y Warley A McCann FE Hider RC Frazer DM Anderson GJ Vulpe CD Simpson RJ McKie AT Identification of an intestinal heme transporter Cell 2005 122 789 801 10.1016/j.cell.2005.06.025 16143108

[B54] Krishnamurthy PC Du GQ Fukuda Y Sun DX Sampath J Mercer KE Wang JF Sosa-Pineda B Murti KG Schuetz JD Identification of a mammalian mitochondrial porphyrin transporter Nature 2006 443 586 589 10.1038/nature05092 17006453

[B55] Krishnamurthy P Xie T Schuetz JD The role of transporters in cellular heme and porphyrin homeostasis Pharmacol Ther 2007 114 345 358 10.1016/j.pharmthera.2007.02.001 17368550

[B56] Galbraith RA McElrath MJ Heme binding to *Leishmania mexicana amazonensis* Mol Biochem Parasitol 1988 29 47 53 10.1016/0166-6851(88)90118-1 3386686

[B57] Lara FA Sant'Anna C Lemos D Laranja GAT Coelho MGP Salles IR Michel A Oliveira PL Cunha-e-Silva N Salmon D Paes MC Heme requirement and intracellular trafficking in *Trypanosoma cruzi *epimastigotes Biochem Biophys Res Commun 2007 355 16 22 10.1016/j.bbrc.2006.12.238 17292866

[B58] Morgan WT Smith A Binding and transport of iron-porphyrins by hemopexin Adv Inorg Chem 2001 51 205 241 full_text

[B59] Tsunoo H Higa Y Kino K Nakajima H Hamaguchi H Studies on hemoglobin metabolism II kinetics of clearance from circulation and hepatic uptake of hemoglobin-haptoglobin complex Proc Jpn Acad Ser B Phys Biol Sci 1977 53 18 21 10.2183/pjab.53.18

[B60] Vincent SH Grady RW Shaklai N Snider JM Mullereberhard U The influence of heme-binding proteins in heme-catalyzed oxidations Arch Biochem Biophys 1988 265 539 550 10.1016/0003-9861(88)90159-2 3421724

[B61] Donohue KV Khalil SM Sonenshine DE Roe RM Heme-binding storage proteins in the Chelicerata J Insect Physiol 2009 55 287 296 10.1016/j.jinsphys.2009.01.002 19183556

[B62] Pascoa V Oliveira PL Dansa-Petretski M Silva JR Alvarenga PH Jacobs-Lorena M Lemos FJA *Aedes aegypti *peritrophic matrix and its interaction with heme during blood digestion Insect Biochem Mol Biol 2002 32 517 523 10.1016/S0965-1748(01)00130-8 11891128

[B63] Gudderra NP Neese PA Sonenshine DE Apperson CS Roe RM Developmental profile, isolation, and biochemical characterization of a novel lipoglycoheme-carrier protein from the American dog tick, *Dermacentor variabilis *(Acari: Ixodidae) and observations on a similar protein in the soft tick, *Ornithodoros parkeri *(Acari: Argasidae) Insect Biochem Mol Biol 2001 31 299 311 10.1016/S0965-1748(00)00122-3 11222939

[B64] Donohue KV Khalil SMS Mitchell RD Sonenshine DE Roe RM Molecular characterization of the major hemelipoglycoprotein in ixodid ticks Insect Mol Biol 2008 17 197 208 10.1111/j.1365-2583.2008.00794.x 18477238

[B65] Maya-Monteiro CM Daffre S Logullo C Lara FA Alves EW Capurro ML Zingali R Almeida IC Oliveira PL HeLp, a heme lipoprotein from the hemolymph of the cattle tick, *Boophilus microplus* J Biol Chem 2000 275 36584 36589 10.1074/jbc.M007344200 10964932

[B66] Gudderra NP Sonenshine DE Apperson CS Roe RM Hemolymph proteins in ticks J Insect Physiol 2002 48 269 278 10.1016/S0022-1910(02)00050-1 12770100

[B67] Hajdušek O Sojka D Kopáček P Burešová V Franta Z Sauman I Winzerling J Grubhoffer L Knockdown of proteins involved in iron metabolism limits tick reproduction and development Proc Natl Acad Sci USA 2009 106 1033 1038 10.1073/pnas.0807961106 19171899 PMC2633537

[B68] Oliveira PL Kawooya JK Ribeiro JMC Meyer T Poorman R Alves EW Walker FA Machado EA Nussenzveig RH Padovan GJ Masuda H A heme-binding protein from hemolymph and oocytes of the bloodsucking insect, *Rhodnius prolixus *- isolation and characterization J Biol Chem 1995 270 10897 10901 10.1074/jbc.270.18.10897 7738030

[B69] Berriman M Haas BJ LoVerde PT Wilson RA Dillon GP Cerqueira GC Mashiyama ST Al-Lazikani B Andrade LF Ashton PD The genome of the blood fluke *Schistosoma mansoni* Nature 2009 460 352 365 10.1038/nature08160 19606141 PMC2756445

[B70] Zylka MJ Reppert SM Discovery of a putative heme-binding protein family (SOUL/HBP) by two-tissue suppression subtractive hybridization and database searches Mol Brain Res 1999 74 175 181 10.1016/S0169-328X(99)00277-6 10640688

[B71] Gobert GN Tran MH Moertel L Mulvenna J Jones MK McManus DP Loukas A Transcriptional changes in *Schistosoma mansoni *during early schistosomula development and in the presence of erythrocytes PLoS Negl Trop Dis 2010 4 2 e600 10.1371/journal.pntd.0000600 20161728 PMC2817720

[B72] Kumar S Guha M Choubey V Maity P Bandyopadhyay U Antimalarial drugs inhibiting hemozoin (beta-hematin) formation: a mechanistic update Life Sci 2007 80 813 828 10.1016/j.lfs.2006.11.008 17157328

[B73] Oliveira MF d'Avila JCP Tempone AJ Soares J Rumjanek FD Ferreira-Pereira A Ferreira ST Oliveira PL Inhibition of heme aggregation by chloroquine reduces *Schistosoma mansoni *infection J Infect Dis 2003 843 852 10.1086/422759 15272414

[B74] Xiao SH Hotez PJ Tanner M Artemether, an effective new agent for chemoprophylaxis against schistosomiasis in China: it *in vivo *effect on the chemical metabolism of the asian schistosome Southeast Asian J Trop Med Public Health 2000 31 724 732 11414420

[B75] De Montellano PRO Wilks A Heme oxygenase structure and mechanism Adv Inorg Chem 2001 51 359 407

[B76] Shibahara S The heme oxygenase dilemma in cellular homeostasis: new insights for the feedback regulation of heme catabolism Tohoku J Exp Med 2003 200 167 186 10.1620/tjem.200.167 14580148

[B77] Okada K The novel heme oxygenase-like protein from *Plasmodium falciparum *converts heme to bilirubin IX alpha in the apicoplast FEBS Lett 2009 583 313 319 10.1016/j.febslet.2008.12.015 19073183

[B78] Deponte M Becker K Glutathione S-transferase from malarial parasites: Structural and functional aspects Methods Enzymol 2005 401 241 253 full_text 16399390 10.1016/S0076-6879(05)01015-3

[B79] Perally S LaCourse EJ Campbell AM Brophy PM Heme transport and detoxification in nematodes: Subproteomics evidence of differential role of glutathione transferases J Proteome Res 2008 7 4557 4565 10.1021/pr800395x 18720983

[B80] Paiva-Silva GO Cruz-Oliveira C Nakayasu ES Maya-Monteiro CM Dunkov BC Masuda H Almeida IC Oliveira PL A heme-degradation pathway in a blood-sucking insect Proc Natl Acad Sci USA 2006 103 8030 8035 10.1073/pnas.0602224103 16698925 PMC1472424

[B81] Srivastava P Puri SK Kamboj KK Pandey VC Glutathione-S-transferase activity in malarial parasites Trop Med Int Health 1999 4 251 254 10.1046/j.1365-3156.1999.00387.x 10320651

[B82] Atamna H Ginsburg H Heme degradation in the presence of glutathione-a proposed mechanism to account for the high-levels of nonheme iron found in the membranes of hemoglobinopathic red-blood-cells J Biol Chem 1995 270 24876 24883 10.1074/jbc.270.42.24876 7559611

[B83] Platel DFN Mangou F Tribouley-Duret J Role of glutathione in the detoxification of ferriprotoporphyrin IX in chloroquine resistant *Plasmodium berghei* Mol Biochem Parasitol 1999 98 215 223 10.1016/S0166-6851(98)00170-4 10080390

[B84] Ginsburg H Famin O Zhang JM Krugliak M Inhibition of glutathione-dependent degradation of heme by chloroquine and amodiaquine as a possible basis for their antimalarial mode of action Biochem Pharmacol 1998 56 1305 1313 10.1016/S0006-2952(98)00184-1 9825729

[B85] Harwaldt P Rahlfs S Becker K Glutathione S-transferase of the malarial parasite *Plasmodium falciparum*: Characterization of a potential drug target Biol Chem 2002 383 821 830 10.1515/BC.2002.086 12108547

[B86] Chen MM Shi LR Sullivan DJ *Haemoproteus *and *Schistosoma *synthesize heme polymers similar to Plasmodium hemozoin and beta-hematin Mol Biochem Parasitol 2001 113 1 8 10.1016/S0166-6851(00)00365-0 11254949

[B87] Pisciotta JM Ponder EL Fried B Sullivan D Hemozoin formation in *Echinostoma trivolvis *rediae Int J Parasitol 2005 35 1037 1042 10.1016/j.ijpara.2005.03.020 16019007

[B88] Oliveira MF d'Avila JC Torres CR Oliveira PL Tempone AJ Rumjanek FD Braga CMS Silva JR Dansa-Petretski M Oliveira MA de Souza W Ferreira ST Haemozoin in *Schistosoma mansoni* Mol Biochem Parasitol 2000 111 217 221 10.1016/S0166-6851(00)00299-1 11087932

[B89] Oliveira MF Gandara ACP Braga CMS Silva JR Mury FB Dansa-Petretski M Menezes D Vannier-Santos MA Oliveira PL Heme crystallization in the midgut of triatomine insects Comp Biochem Physiol C Toxicol Pharmacol 2007 146 168 174 10.1016/j.cbpc.2006.12.007 17254848

[B90] Oliveira MF Silva JR Dansa-Petretski M de Souza W Braga CMS Masuda H Oliveira PL Haemozoin formation in the midgut of the blood-sucking insect *Rhodnius prolixus* FEBS Lett 2000 477 95 98 10.1016/S0014-5793(00)01786-5 10899317

[B91] Correa Soares JB Menezes D Vannier-Santos MA Ferreira-Pereira A Almeida GT Venancio TM Verjovski-Almeida S Zishiri VK Kuter D Hunter R Egan TJ Oliveira MF Interference with hemozoin formation represents an important mechanism of schistosomicidal action of antimalarial quinoline methanols PLoS Negl Trop Dis 2009 3 e477 10.1371/journal.pntd.0000477 19597543 PMC2703804

[B92] Brown WH Malarial pigment (so-called melanin): Its nature and mode of production J Exp Med 1911 13 290 299 10.1084/jem.13.2.290 19867409 PMC2124860

[B93] Fitch CD Kanjananggulpan P The state of ferriprotoporphyrin-IX in malaria pigment J Biol Chem 1987 262 15552 15555 3119578

[B94] Slater AFG Swiggard WJ Orton BR Flitter WD Goldberg DE Cerami A Henderson GB An iron carboxylate bond links the heme units of malaria pigment Proc Natl Acad Sci USA 1991 88 325 329 10.1073/pnas.88.2.325 1988933 PMC50803

[B95] Pandey AV Tekwani BL Formation of haemozoin/beta-haematin under physiological conditions is not spontaneous FEBS Lett 1996 393 189 193 10.1016/0014-5793(96)00881-2 8814287

[B96] Tripathi AK Khan SI Walker LA Tekwani BL Spectrophotometric determination of de novo hemozoin/beta-hematin formation in an in vitro assay Anal Biochem 2004 325 85 91 10.1016/j.ab.2003.10.016 14715288

[B97] Pagola S Stephens PW Bohle DS Kosar AD Madsen SK The structure of malaria pigment beta-haematin Nature 2000 404 307 310 10.1038/35005132 10749217

[B98] Wood BR Langford SJ Cooke BM Lim J Glenister FK Duriska M Unthank JK McNaughton D Resonance Raman spectroscopy reveals new insight into the electronic structure of beta-hematin and malaria pigment J Am Chem Soc 2004 126 9233 9239 10.1021/ja038691x 15281812

[B99] Hoang AN Ncokazi KK de Villiers KA Wright DW Egan TJ Crystallization of synthetic haemozoin (beta-haematin) nucleated at the surface of lipid particles Dalton Trans 2010 39 1235 1244 10.1039/b914359a 20104349 PMC2889375

[B100] Slater AFG Cerami A Inhibition by chloroquine of a novel heme polymerase enzyme-activity in malaria trophozoites Nature 1992 355 167 169 10.1038/355167a0 1729651

[B101] Dorn A Stoffel R Matile H Bubendorf A Ridley RG Malarial haemozoin/beta-haematin supports haem polymerization in the absence of protein Nature 1995 374 269 271 10.1038/374269a0 7885447

[B102] Egan TJ Recent advances in understanding the mechanism of hemozoin (malaria pigment) formation J Inorg Biochem 2008 102 1288 1299 10.1016/j.jinorgbio.2007.12.004 18226838

[B103] Pandey AV Babbarwal VK Okoyeh JN Joshi RM Puri SK Singh RL Chauhan VS Hemozoin formation in malaria: a two-step process involving histidine-rich proteins and lipids Biochem Biophys Res Commun 2003 308 736 743 10.1016/S0006-291X(03)01465-7 12927780

[B104] Jani D Nagarkatti R Beatty W Angel R Slebodnick C Andersen J Kumar S Rathore D HDP-A novel heme detoxification protein from the malaria parasite PLoS Pathog 2008 4 15 10.1371/journal.ppat.1000053 PMC2291572 18437218

[B105] Tellam RL Wijffels G Willadsen P Peritrophic matrix proteins Insect Biochem Mol Biol 1999 29 87 101 10.1016/S0965-1748(98)00123-4 10196732

[B106] Lehane MJ Peritrophic matrix structure and function Annu Rev Entomol 1997 42 525 550 10.1146/annurev.ento.42.1.525 15012322

[B107] Devenport M Alvarenga PH Shao L Fujioka H Bianconi ML Oliveira PL Jacobs-Lorena M Identification of the *Aedes aegypti *peritrophic matrix protein AeIMUCI as a heme-binding protein Biochemistry 2006 45 9540 9549 10.1021/bi0605991 16878988

[B108] Lara FA Lins U Paiva-Silva G Almeida IC Braga CM Miguens FC Oliveira PL Dansa-Petretski M A new intracellular pathway of haem detoxification in the midgut of the cattle tick *Boophilus microplus*: aggregation inside a specialized organelle, the hemosome J Exp Biol 2003 206 1707 1715 10.1242/jeb.00334 12682102

[B109] Glanfield A McManus DP Anderson GJ Jones MK Pumping iron: a potential target for novel therapeutics against schistosomes Trends Parasitol 2007 23 583 8 10.1016/j.pt.2007.08.018 17962074 PMC2756500

